# Supplementary material for: Intracavity optical trapping of microscopic particles in a ring-cavity fiber laser
Source: Nat Commun. 2019 Jun 18;10:2683. doi: 10.1038/s41467-019-10662-7 (PMC6581956; doi:10.1038/s41467-019-10662-7)
Supplement: Supplementary file 1 — Supplementary Information [file 41467_2019_10662_MOESM1_ESM.pdf]

Supplementary Information

Intracavity Optical Trapping of Microscopic Particles in a Ring-Cavity Fiber Laser

Kalantarifard *et al.*

Technique	Sketch	Objective, Lens	Particle	Diameter (μm)	I (mW μm^{-2})	k I^{-1} (pN μm mW^{-1})	Reference
OT	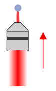	High NA, ~ 1.3	PS	4.9	2.3	0.026	This work
CPOT	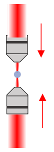	Low NA, ~ 0.65	PS	4.0	10.0	0.200	Woerdemann *et al.,* Opt. Express (2010)
MT	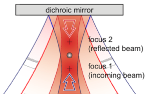	Low NA, ~ 0.2	PS	4.5	0.14	1.42	Pitzek *et al.,* Opt. Express (2009)
SIBA	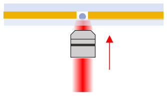	Low NA, ~ 0.65	PS	0.1	1.0	0.01	Juan *et al.,* Nat. Phys. (2009)
ICOT	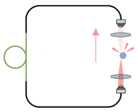	Low NA, ~ 0.12	PS	4.9	0.012	9.16	This work

Supplementary Figure 1. **Comparison of different optical trapping schemes.** We compare the intracavity nonlinear-feedback trapping scheme to standard optical tweezers (OT) using a high numerical aperture objective (NA=1.30) and trapping the same polystyrene particle with a diameter of 4.9 µm, as well as to other optical trapping schemes proposed in the literature, namely counterpropagating beam trapping (CPOT) (aka dual-beam optical trapping) [1], mirror optical trapping (MT) [2], and SIBA trapping [3]. For a quantitative comparison, we consider the light intensity at the sample, I, and the trap stiffness per unit intensity, κI^{-1}, for polystyrene particles of similar size (when possible). Note how the intracavity optical trapping scheme has the lowest intensity at the sample with the highest stiffness per unit intensity.

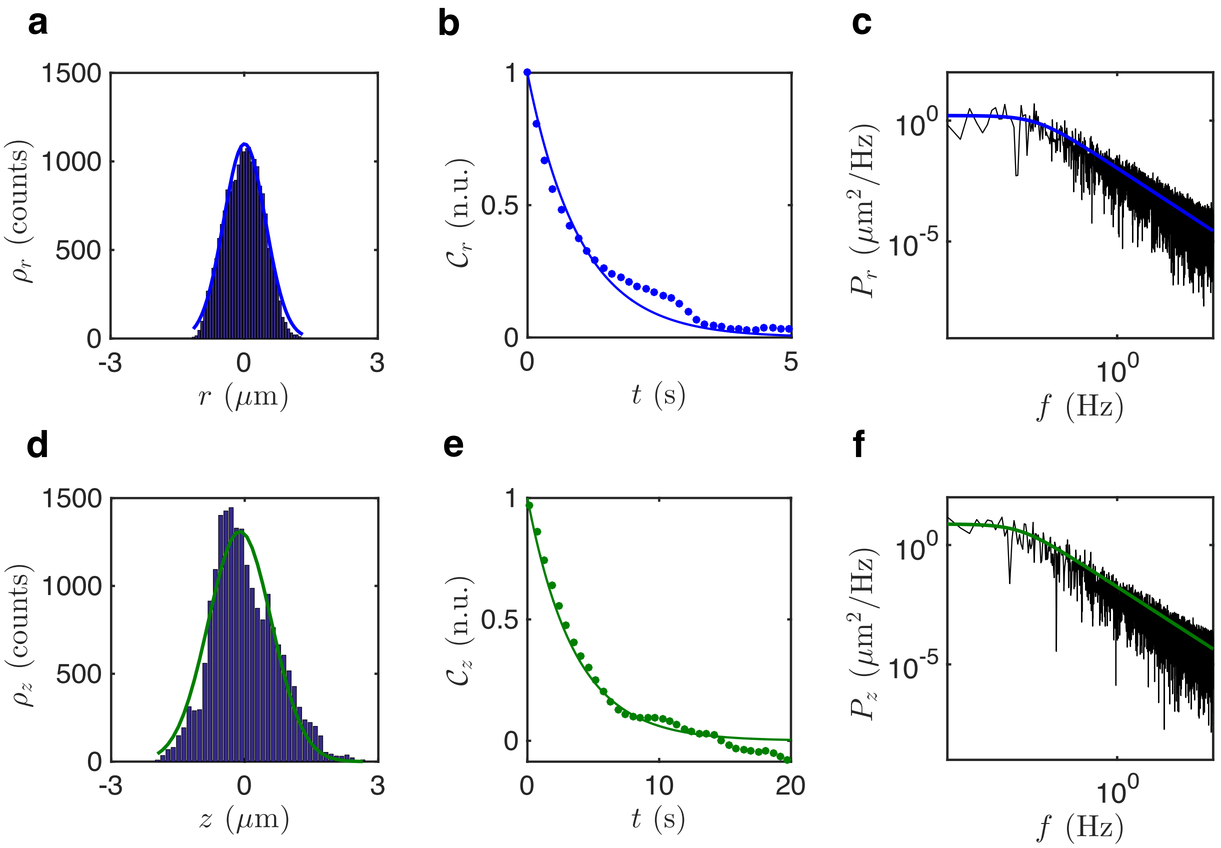

Supplementary Figure 2. **Analysis of the simulation results.** **a** Probability distributions, **b** position autocorrelation functions (ACFs), and **c** power spectral densities (PSDs) along the transverse x-direction for a simulated trajectory of a 4.9-µm-diameter polystyrene particle held in the intracavity optical trap (corresponding to the results shown in Figure 3). **d** Probability distributions, **e** ACFs and **f** PSDs along the axial z-direction. The solid lines are the best fit to the data from which we calculate the trap stiffnesses of the simulated intracavity trap. These analyses are consistent with a Hookean force profile for small displacements ($r < 1\,\mu$m) and short times ($t < 1\,$s), which permits us to perform a meaningful calibration of the optical trapping forces using standard techniques.

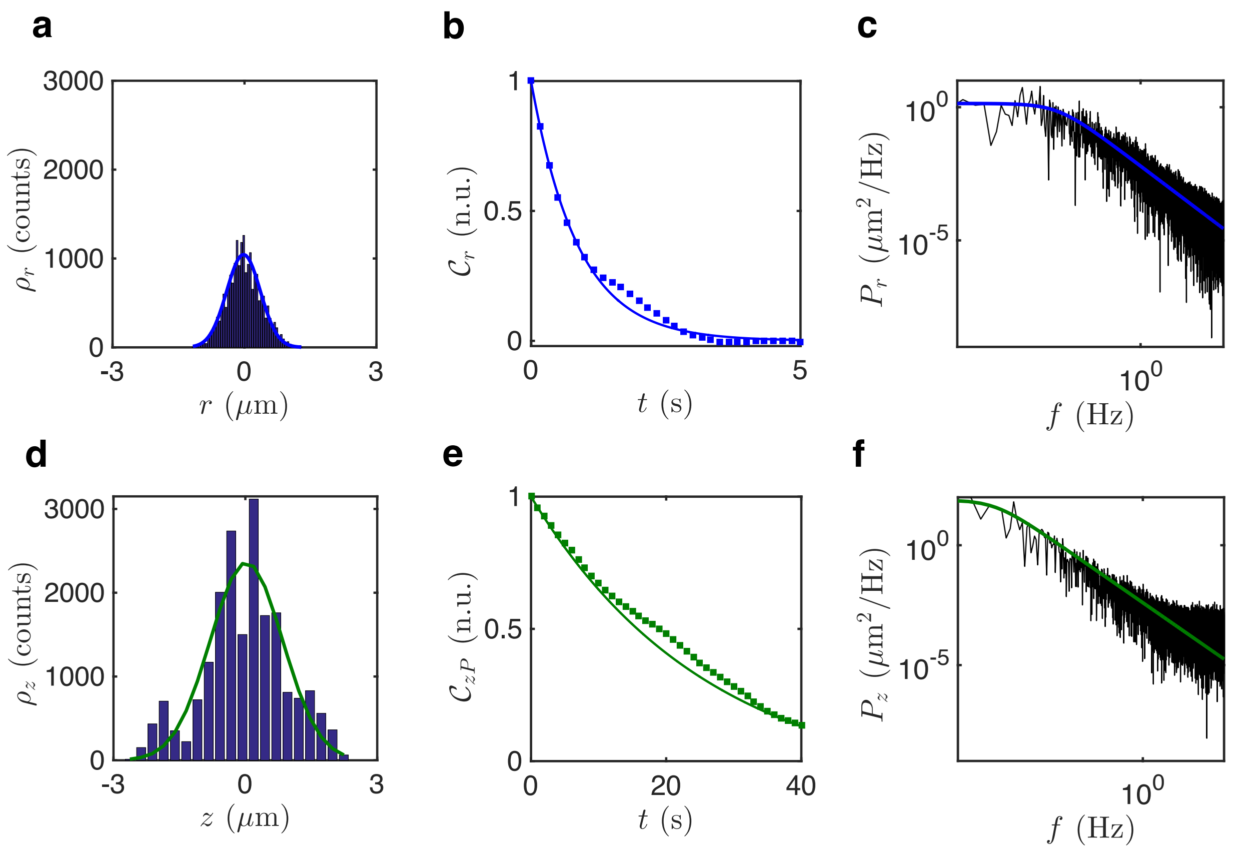

Supplementary Figure 3. **Analysis of the experimental results. a** Probability distributions, **b** position autocorrelation functions (ACFs), and **c** power spectral densities (PSDs) along the transverse x-direction for an experimental trajectory of a 4.9-μm-diameter polystyrene particle held in the intracavity optical trap (corresponding to the results shown in Figure 5). **d** Probability distributions, **e** ACFs and **f** PSDs along the axial z-direction. The solid lines are the best fit to the data from which we calculate the trap stiffnesses of the simulated intracavity trap. These results are in agreement to those shown in Supplementary Figure 2. These analyses are consistent with a Hookean force profile for small displacements ($r < 1$ μm) and short times ($t < 1$ s), which permits us to perform a meaningful calibration of the optical trapping forces using standard techniques.

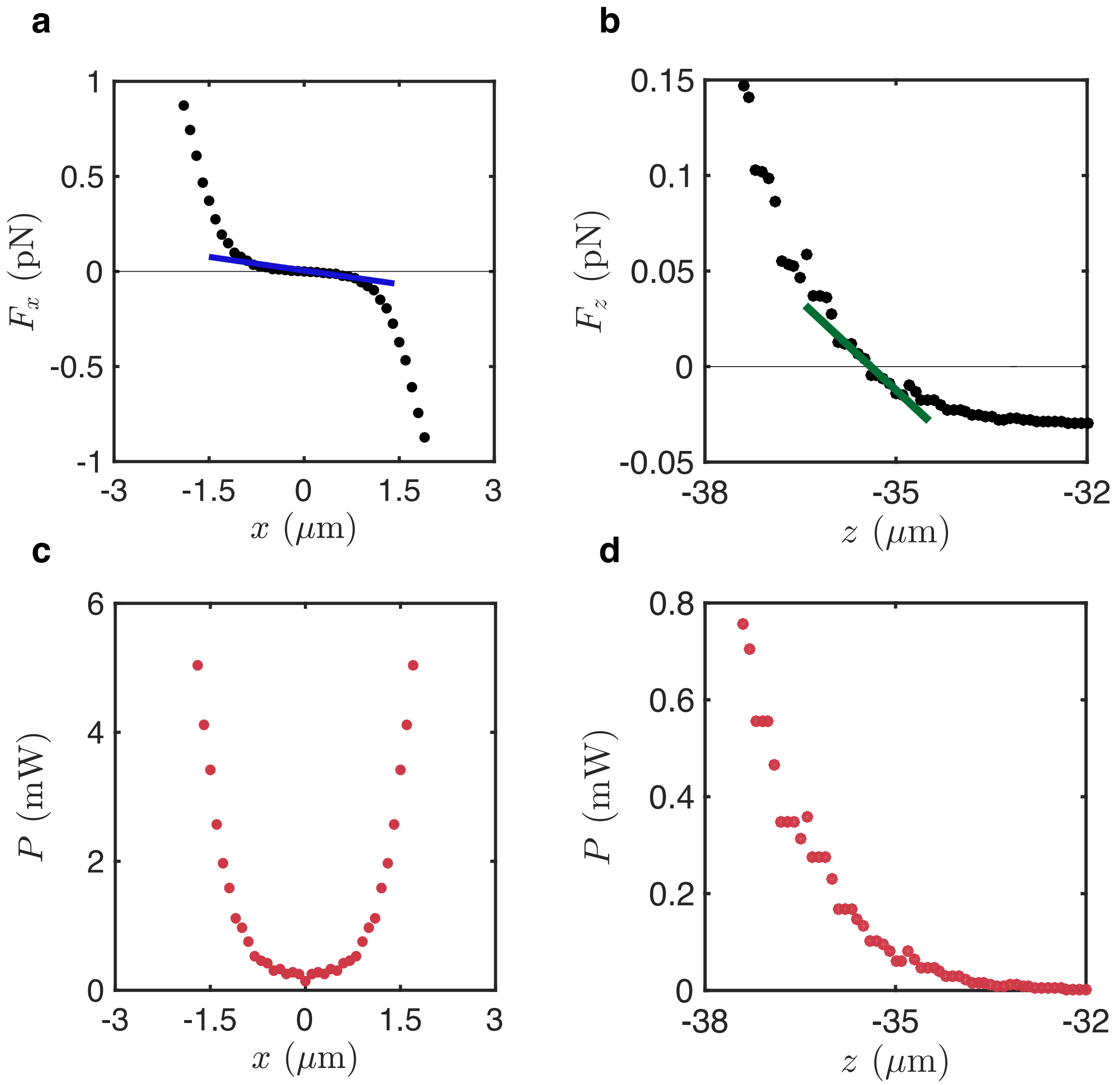

Supplementary Figure 4. **Force and power versus displacement. a-b** Simulations of the radial and axial force, and **c-d** corresponding intracavity power for a 4.9-µm-diameter polystyrene particle displaced from its equilibrium position. The solid lines are linear fits that show the linearity of the force for small displacements, i.e., $F_x \approx -k_x x$ and $F_z \approx -k_z z$. For large displacements the nonlinear behaviour dictated by the increased optical feedback related to the reduction of scattered light is clearly visible.

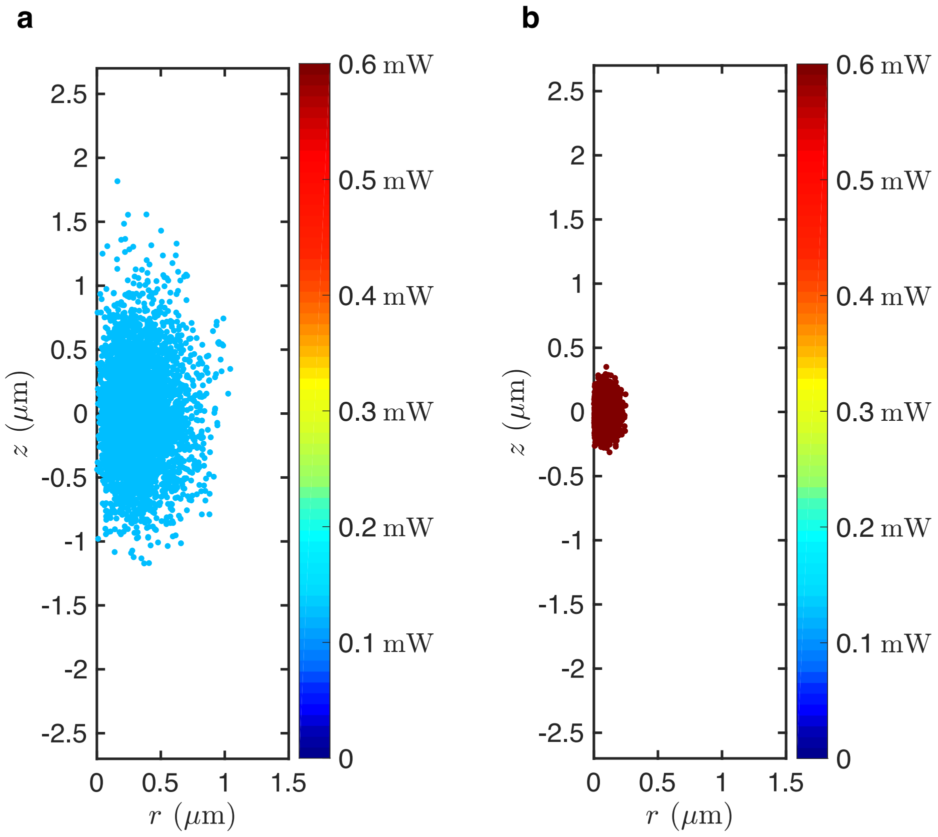

Supplementary Figure 5. **Uncorrelated power-position maps in standard optical tweezers using a high-NA objective.** Simulations of a high-NA optical trap operating **a** at a power of 0.12 mW corresponding to the intracavity average trap power (note that a real high-NA trap with such power is not able to trap a microscopic particle, because the particle escapes from the central region of the trap where the restoring force is harmonic, while in the simulation we assume for illustration purposes the harmonic trapping region to extend to infinity) and **b** at a power of 3 mW that is close to the trapping threshold in experiments. The trap power is decoupled from the particle fluctuations and hence the colour map is constant at the specified value. The positional fluctuations follow the Gaussian distribution typical of an optical tweezers that for small displacements is well approximated by a harmonic potential. Note that in both cases the intensity of laser light at sample is about two orders of magnitude more than that of the intracavity trap.

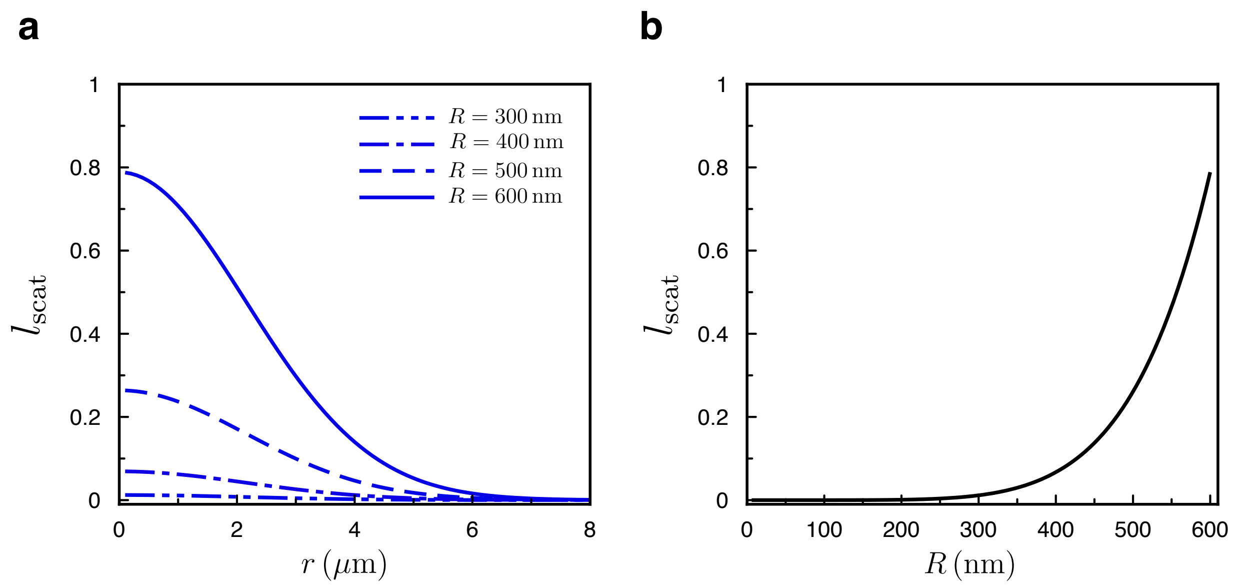

Supplementary Figure 6. **Intracavity optical loss for small particles. a** Optical loss as a function of displacement calculated for our experimental parameters (NA=0.12, $\lambda_0 = 1030\,\mathrm{nm}$) in the dipole approximation for polystyrene particles with radii of 300, 400, 500, and 600 nm. The optical loss profile follows the Gaussian intensity profile of the incident laser beam. **b** Size scaling of the optical loss in the dipole approximation for polystyrene particles. Since the particles are non-absorbing the scaling rapidly decreases as R^6 for small radii. This clearly shows that, for our experimental parameters, the intracavity feedback trapping is efficient at the microscale while it reduces to a standard single-beam optical trapping at the nanoscale.

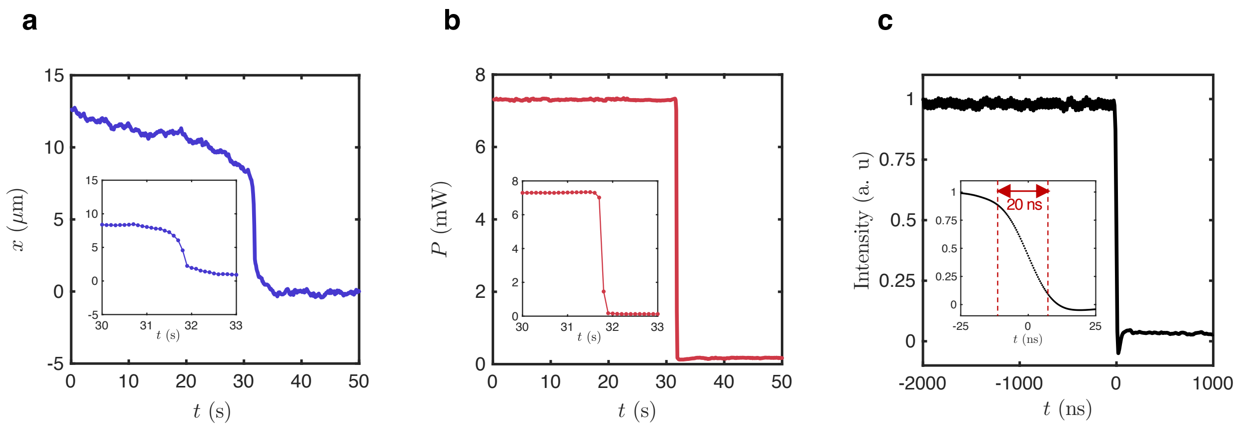

Supplementary Figure 7. Temporal dynamics of intracavity optical trapping and laser cavity. Temporal dynamics of a 4.9-μm-diameter polystyrene particle during intracavity trapping. The particle position is tracked **a** while the particle reaches its equilibrium position and **b** as the particle is trapped the ring-laser dynamics changes and intracavity power drops to the low-level value corresponding to the increased optical losses. The slopes of both the particle position and power drop correspond to about 100 ms, clearly showing how the particle position directly affect the laser operation. The insets in **a** and **b** represent the position and corresponding power for a shorter time scale where the particle is falling into the trap. **c** Measured response of the laser cavity to a dynamical loss produced using an AOM (acousto-optic modulator) inside the cavity. The inset represents the zoomed in data where the signal drops. As shown in the inset, the $10-90\%$ response time of the laser is about 20 ns.

SUPPLEMENTARY NOTE 1

Comparison of intracavity optical trapping with other optical trapping schemes

In the main text, we directly compare our intracavity nonlinear-feedback trapping scheme to standard single-beam optical trapping, i.e., standard optical tweezers, using a high numerical aperture objective (NA=1.3) and trapping the same polystyrene particle with a diameter of 4.9 μm. Here, we compare it with other examples of optical trapping schemes in the literature, namely, counterpropagating beam trapping or dual-beam optical trapping [1], mirror optical trapping [2], and self-induced back action (SIBA) trapping [3, 4]. In these trapping techniques a low NA objective lens is generally used and both nanoparticles and microparticles can be confined with a relatively low intensity of laser light at the sample. Clearly each technique has its own positive and negative aspects. For example, SIBA trapping can efficiently trap nanoparticles, but is intrinsically working in close proximity with a substrate. On the other hand, counterpropagating beam and mirror trapping are specifically designed to remove radiation pressure increasing trap stability, although alignment of the beams is more critical than in other schemes. We stress that intracavity optical trapping is essentially different from other approaches as it is an all-optical nonlinear feedback scheme that exploit a single-beam in an active laser cavity. Since particle-light interaction is crucially dependent on particle size and composition (e.g., dielectric, metallic), for a fair quantitative comparison of the different techniques in Supplementary Figure 1, we consider parameters such as the intensity at the sample and the trap stiffness per unit intensity for polystyrene particles of similar size (when possible).

Self induced back action trap: This is a feedback trap [3, 4] where the position of a nanoparticle influences the strength of the trap dictated by a passive plasmonic nanocavity. Generally it uses a nanoaperture in a metal film that is close to its cutoff resonance. Thus, the feedback trapping mechanism relies on the presence of the particle to decrease the local electric field by shifting the plasmonic cavity mode away from the incident wavelength. This technique achieves optical trapping of 100 nm and 50 nm polystyrene spheres with local intensity of about $1\,\mathrm{mW\,\mu m^{-2}}$. This technique is perfectly suited for the nanoscale and performs better as the particle size is reduced while the trapping time decreases as the size of particle is increased. It is worth noting that in this scheme, as for our intracavity scheme,

the lateral trapping force is linear for small displacement and becomes nonlinear for large displacements.

Counter propagating beam trap: This method, originally exploited in 1970 by Arthur Ashkin [5] to stably trap particles in liquid, is based on two counter propagating beams focused by low NA objectives that provide three-dimensional trapping resulting from the cancelation of the longitudinal scattering forces. In reference [1] a method that provides counterpropagating optical trapping using optical phase-conjugation is presented. The experiment exploits a photo refractive phase-conjugate mirror to create a back-propagating beam that is automatically matched with an arbitrary incoming beam. By this means single and multiple trapping is achieved. In the case of single particle, a 4 μm polystyrene particle is trapped at low power and low intensity (about $10\,\mathrm{mW}\,\mu\mathrm{m}^{-2}$). However, the longitudinal potential is relatively weak, so after axial displacement, the particle needs a few seconds to return to the trapping position in focus. This method also suffers from dependent trapping and imaging as two objectives are used for creating counter propagating beams and the imaging path cannot be separated from the trapping path. A possibility is also to exploit this method in a capillary, where the imaging system can be placed orthogonally to the beam path [6].

Mirror trap: This technique is an evolution of the dual beam trap in which two collinear beams are shaped by a spatial light modulator. The first beam is focused before a planar mirror, while the second beam is focused after reflection from a mirror. This method realizes three-dimensional trapping using a single low NA objective. By means of mirror trapping, optical trapping of polystyrene microparticles with diameters from 1.4 μm to 45 μm have been demonstrated [2]. The estimated laser intensity at the sample for a particle of 4.5 μm is about about $0.14\,\mathrm{mW}\,\mu\mathrm{m}^{-2}$ (see Supplementary Figure 1).

SUPPLEMENTARY NOTE 2

Analysis of the optical potential generated by the intracavity optical trap

We have analyzed the effective optical potential generated by the intracavity optical trap using the trajectories of an optically trapped particle obtained from simulations (Supplementary Figure 2) and experiments (Supplementary Figure 3). We have analyzed these

trajectories using three standard methods (see below), which are based on the Langevin description of a Brownian particle in thermal equilibrium and give a direct calibration of the trap stiffness of the effective optical potential [7, 8].

The results of all analyses are consistent with an effective harmonic confinement for small displacements around the equilibrium position and short times. Thus, we can meaningfully measure the associated transverse and axial force constants. However, we remark that deviations emerge for large displacements and long times. See also results shown in Supplementary Figure 4 and Supplementary Note 3.

Potential analysis. We start our analysis by considering the Langevin equation which describes the motion of a particle in an effective external potential, $U(x)$, and subject to a random thermal force. In the low-Reynolds-number regime, applicable for microparticles suspended in water, it is possible to drop the inertial term obtaining the overdamped Langevin equation:

$$\frac{dx(t)}{dt} + \frac{1}{\gamma}\frac{d}{dx}U(x) = \xi_x(t) \tag{1}$$

where γ is the friction coefficient, $\xi(t) = \sqrt{2D}W(t)$ is a white noise with zero mean (i.e., while $\langle\xi(t)\rangle = 0$) and intensity $2D$ (i.e., while $\langle\xi(t)^2\rangle = 2D$), and D is the diffusion coefficient. As Brownian particles are in thermal equilibrium, the probability distribution of these particles in the fluid is given by the Maxwell-Boltzmann distribution:

$$\rho(x) = \rho_0 \exp\left[-\frac{U(x)}{k_B T}\right], \tag{2}$$

where ρ_0 is a constant factor coming from normalization.

For small displacements, an optically trapped particle is confined in a harmonic potential with spring constant k_x, i.e., $U(x) = \frac{1}{2}k_x(x - x_{eq})^2$. Thus, the corresponding Langevin equation becomes:

$$\frac{dx(t)}{dt} = -\frac{k_x}{\gamma}x(t) + \sqrt{2D}W_x(t) \tag{3}$$

and the particle probability distribution is Gaussian:

$$\rho(x) = \rho_0 \exp\left[-\frac{k_x(x - x_{eq})^2}{2k_B T}\right]. \tag{4}$$

We measure the histograms of the particle position from the particle trajectories obtained from simulations and experiments for a 4.9-μm-diameter polystyrene particle trapped in the intracavity optical tweezers. In detail, we plot the distribution of the positions of the particle,

x_i (with $i = 1, ..., I$) as a series of independent samples acquired at t_i for time duration T. The samples are arranged into a series along the x direction, such that a_n shows how many of the positions fall within the n-th bin. The histogram a_n is an approximation of the probability distribution for position, $\rho(x)$. The numerical results are plotted by the histograms in Supplementary Figures 2(a) for the x-direction and 2(d) for the z-direction; the corresponding experimental results are plotted in Supplementary Figures 3(a) and 3(d). These results are then fitted to the theoretical particle probability distribution given by Eq. (4) (solid lines in Supplementary Figures 2(a), 2(d), 3(a), and 3(d)).

From the histogram of the particle probability distribution, we estimate the particle equilibrium position as:

$$x_{\rm eq} = \frac{1}{I}\sum_{i=1}^{N} x_i, \tag{5}$$

the position variance as:

$$\sigma_x^2 = \frac{1}{I}\sum_{i=1}^{N}(x_i - x_{\rm eq})^2, \tag{6}$$

and the corresponding trap stiffness as :

$$k_x^{\rm exp} = \frac{k_B T}{\sigma_x^2}. \tag{7}$$

We remark that the last formula is only strictly valid for a harmonic potential.

From this analysis we can observe that the particle probability distributions obtained for the particle trapped by the intracavity optical trap are well-approximated by a Gaussian around the equilibrium position, while some deviations emerge in the tails, where the nonlinearity becomes evident in the sub-Gaussian tails of the probability distributions.

Correlation analysis. We start by considering the generic expression of the positional autocorrelation function (ACF):

$$C_x(\tau) = \langle x(t)x(t+\tau)\rangle \tag{8}$$

and its derivative with respect to a lag time τ:

$$\frac{dC_x(\tau)}{d\tau} = \left\langle x(t)\frac{dx(t+\tau)}{d\tau}\right\rangle. \tag{9}$$

We now consider the overdamped Langevin equation (Eq. (1)) at $t = t+\tau$, multiply by $x(t)$, and then time average. Since $x(t)$ and $\xi_x(t)$ are uncorrelated functions, we obtain a simple

differential equation for the correlation function:

$$\frac{dC_x(\tau)}{d\tau} = -\frac{k_x}{\gamma} C_x(\tau), \tag{10}$$

which has an exponential solution:

$$C_x(\tau) = \frac{k_B T}{k_x} e^{-\frac{k_x}{\gamma}\tau}. \tag{11}$$

This expression is used to calibrate the optical trapping potential from autocorrelation functions obtained from the simulated or experimental particle trajectories.

We measure the ACF from the particle trajectories obtained from simulations and experiments for a 4.9-μm-diameter polystyrene particle trapped in the intracavity optical tweezers. To do so, we need a time series of correlated positions in several time intervals, i.e., sample positions $x_i = x(t_i)$ where $i = 1, ..., N$, at times $t_i = i\Delta t$. Then the ACF can be obtained from the trajectory at time lags $t_j = j\Delta t$ as:

$$C_{x,j} = \frac{1}{N-j} \sum_{i=1}^{N-j} x_{i+j} x_i. \tag{12}$$

By fitting these ACF obtained from simulated and experimental trajectories, it is possible to obtain the trap constants. Repeating the analysis for a number of experimental series, an average value for k_x and also a standard deviation can be calculated.

The numerical results are plotted by the dots in Supplementary Figures 2(b) for the x-direction and 2(e) for the z-direction; the corresponding experimental results are plotted in Supplementary Figures 3(b) and 3(e). The corresponding fitting to the theoretical ACF (Eq. (11)) are given by the solid lines in Supplementary Figures 2(b), 2(e), 3(b), and 3(e).

From these results we can observe that the ACFs of the particle trapped by the intracavity optical trap are well approximated by the exponential decay typical of a particle trapped in a harmonic potential, while some deviations appear at intermediate times.

Power spectrum analysis. The starting point is always the overdamped Langevin equation of motion of an optically trapped particle given in one dimension. Introducing the corner frequency, $f_c = \frac{k_x}{2\pi\gamma}$, we obtain the following Langevin equation:

$$\frac{dx(t)}{dt} + 2\pi f_c x(t) = \sqrt{2D} W(t). \tag{13}$$

Its Fourier transform is:

$$-2\pi i f \tilde{x}(f) + 2\pi f_c \tilde{x}(f) = \sqrt{2D} \tilde{W}(f) \tag{14}$$

where $\tilde{x}(f)$ and $\tilde{W}(f)$ are the Fourier transform of $x(t)$ and $W(t)$. Since $|\tilde{W}(f)|^2 \equiv 1$, taking the square modulus of both sides of the equation, we obtain the power spectral density (PSD) of the particle motion as:

$$|\tilde{x}(f)|^2 = \frac{D}{2\pi^2(f^2 + f_{\mathrm{c}}^2)}. \tag{15}$$

This PSD has a Lorentzian shape with the half-width half-maximum that is the relaxation frequency of trap, f_{c}. At zero frequency, the power spectrum presents a value of $D(2\pi^2)^{-1}f_{\mathrm{c}}^{-2}$, related to the particle's diffusion and trap spring constant.

We measure the PSD from the particle trajectories obtained from simulations and experiments for a 4.9-µm-diameter polystyrene particle trapped in the intracavity optical tweezers. To do so, we sample the trajectory at regular time intervals with frequency f_{s}. Therefore we obtain the sample positions $x_i = x(t_i)$ with $i = 1, ..., N$ at the time intervals $t_i = i\Delta t$. where Δt is the sampling time step $\Delta t = f_{\mathrm{s}}^{-1}$. The finite difference equation corresponding to the overdamped Langevin equation is:

$$x_{i+1} = x_i - 2\pi f_{\mathrm{c}} x_i \Delta t + \sqrt{2D\Delta t} w_i, \tag{16}$$

where w_i are Gaussian random numbers with zero mean and unitary variance. The corresponding Fourier transform in discrete form is:

$$\tilde{x}_j = \Delta t \sum_{i=1}^{N} e^{i2\pi f_j t_i} x_i, \tag{17}$$

where $j = -\frac{N}{2} + 1, ..., \frac{N}{2}$. By substituting $f_j = \frac{j}{t_s}$ in the above expression, we get:

$$\tilde{x}_j = \Delta t \sum_{i=1}^{N} e^{i2\pi ij/N} x_i. \tag{18}$$

Using this expression in the finite difference equation and making a simplification to approxiamte $e^{i2\pi j/N}$ for $j \ll N$, this results in:

$$\frac{|\tilde{x}(f)|^2}{\sqrt{t_s}} = \frac{D}{2\pi^2 \Delta t_s (f_j^2 + f_{\mathrm{c}}^2)} |\tilde{W}_j|^2, \tag{19}$$

with the expected values of the PSD:

$$\langle P \rangle = \left\langle \frac{|\tilde{x}(f)|^2}{\sqrt{t_s}} \right\rangle = \frac{D}{2\pi^2 \Delta t_s (f_j^2 + f_{\mathrm{c}}^2)}. \tag{20}$$

By fitting the power spectrum of the tracked positions, it is possible to calculate the corner frequency and trap constants.

The numerical results are plotted by the black line in Supplementary Figures 2(c) for the x-direction and 2(f) for the z-direction; the corresponding experimental results are plotted in Supplementary Figures 3(c) and 3(f). The corresponding fitting to the theoretical PSD (Eq. (15)) are given by the solid lines in Supplementary Figures 2(c), 2(f), 3(c), and 3(f).

From these results we can observe that the PSDs of the particle trapped by the intracavity optical trap are well approximated by the Lorentzian shape typical of a particle trapped in a harmonic potential.

SUPPLEMENTARY NOTE 3

Nonlinearity of the displacement-force curves

As discussed in the main text, a toy model can be used to give a qualitative description of intracavity optical trapping. In this simple picture the power and force are zero over some range of displacements, and then a nonlinear force is exerted out of this range. This model is substantially confirmed by the full accurate simulation described in the manuscript as shown along the transverse x-direction in Supplementary Figures 4(a) and 4(c): the force is linear for small displacements consistent with a Hookean response of the trap (Supplementary Figure 4(a)); and the power on the particle is very small for small displacements. The corresponding results for the z-direction are shown in Supplementary Figures 4(b) and 4(d). It can be clearly seen (solid lines) that for small displacements the force versus position has a linear region for both transverse and axial directions. However, for larger displacements, a transition from a linear to a nonlinear regime is observed. The corresponding power in the transverse direction (Supplementary Figures 4(a) and 4(c)) is below the laser threshold in the linear regime for small displacements and increases suddenly for large displacements within the nonlinear regime. In the axial direction (Supplementary Figures 4(b) and 4(d)), whenever the particle sags due to gravity, the power increases and pushes it up towards the equilibrium position. If the particle is pushed up too much, the power decreases letting the particle move downwards towards the trap equilibrium position.

SUPPLEMENTARY NOTE 4

Positional fluctuations and intensity in the standard high-NA optical tweezers

In Figure 3 of the main text we plot the intensity map relating the particle positional fluctuations with intracavity power. Here we show complementary results to compare the positional fluctuations in our standard high NA (1.3) optical tweezers. Supplementary Figure 5(a) shows ray optics simulations for a 4.9-μm-diameter polystyrene particle in a high NA trap operating at a power of about 0.12 mW similar to the average operating power used for intracavity trapping simulations. Supplementary Figure 5(b) shows ray optics simulations corresponding to a power of 3 mW that is close to the trapping threshold in high NA optical tweezers experiments. In a standard optical tweezers power and particle position are decoupled since there is no feedback. Thus, the power and intensity are constant, and the colour maps are represented with a single colour corresponding to this constant value. The positional fluctuations follow the Gaussian distribution typical of a high NA optical tweezers that, for small displacements, is well approximated by a harmonic potential. Note that the actual threshold power for stable trapping in experiments (Figure 5(b)) is much higher (at least one order of magnitude) than the intracavity case clarifying that standard optical tweezers with high NA requires much more intensity to operate while intracavity trapping can work efficiently at low power. Finally, we note that ray optics simulations have a lower trapping threshold than experiments. This is because ray optics overestimate the axial trapping force that appears weaker in experiments [9, 10]. The theory does not take into account effects such as aberrations or distortions from the two glass-water interfaces that can play an important role in weakening the trapping stiffness in experiments. This discrepancy is also related to the lower axial stiffness measured in experiments and shown in Figure 6 in the main text.

SUPPLEMENTARY NOTE 5

Intracavity optical trapping for small particles

In order to gain some analytical insight into intracavity optical trapping for small particles, we can examine how it behaves in the limit of the dipole approximation. In particular,

we can shed light on the relation between extinction cross section, particle displacement, scattered power, and cavity losses as well as describe the size scaling behavior of such quantities for small particles. Note that for small particles the power scattered in the trap decreases and hence the nonlinear feedback that regulates intracavity trapping will be reduced. For negligible losses, i.e., $l_{\mathrm{scat}} \approx 0$, the intracavity trap behaves as a standard single-beam optical tweezers.

When the particle size parameter is small, i.e., $x \ll 1$, optical trapping forces can be calculated exploiting the dipole approximation, i.e., the particle can be approximated as a small induced dipole immersed in an electromagnetic field $\mathbf{E}(\mathbf{r}, t)$, which can be considered homogeneous inside the particle ($x|n_{\mathrm{p}}/n_{\mathrm{m}}| \ll 1$) [11–13]. If the external field is not too large, the induced dipole moment, $\mathbf{p}(\mathbf{r}, t)$, can be expressed in terms of a linear complex polarizability, α_{p}, with respect to the surrounding medium, given by Draine and Goodman [14]:

$$\alpha_{\mathrm{p}} = \alpha_0 \left(1 - i\frac{k_{\mathrm{m}}^3 \alpha_0}{6\pi\varepsilon_{\mathrm{m}}}\right)^{-1} \tag{21}$$

with ε_{m} being the dielectric permittivity of the medium and α_0 being the static Clausius-Mossotti polarizability, $\alpha_0 = 3V\varepsilon_{\mathrm{m}} \left(\varepsilon_{\mathrm{p}} - \varepsilon_{\mathrm{m}}\right) / \left(\varepsilon_{\mathrm{p}} + 2\varepsilon_{\mathrm{m}}\right)$, where V is the particle volume and ε_{p} the dielectric permittivity of the particle. In this regime, the polarizability is also related to the cross-sections: the light-particle interaction, including optical forces, can be described in terms of extinction, σ_{ext}, scattering, σ_{scat}, and absorption, $\sigma_{\mathrm{abs}} = \sigma_{\mathrm{ext}} - \sigma_{\mathrm{scat}}$, cross sections. In particular, for a small particle of polarizability α_{p}, we can write the extinction and scattering cross-sections as [7]:

$$\sigma_{\mathrm{ext,d}} = \frac{k_{\mathrm{m}}}{\varepsilon_{\mathrm{m}}} \Im\{\alpha_{\mathrm{p}}\}, \qquad \sigma_{\mathrm{scat,d}} = \frac{k_{\mathrm{m}}^4}{6\pi\varepsilon_{\mathrm{m}}^2} |\alpha_{\mathrm{p}}|^2. \tag{22}$$

Then, we can consider the time-averaged optical force experienced by a small particle when illuminated by a time-varying electromagnetic field [12, 13, 15]:

$$\mathbf{F}_{\mathrm{DA}} = \frac{1}{2} \Re \left\{ \sum_i \alpha_{\mathrm{p}} E_i \nabla E_i^* \right\}, \tag{23}$$

where E_i are the electric field components. Starting from this expression, one can explicitly write the optical force in terms of extinction cross-section and particle's polarizability [12, 13, 15–17]:

$$\mathbf{F}_{\mathrm{DA}} = \frac{1}{2} \frac{n_{\mathrm{m}}}{c\varepsilon_{\mathrm{m}}} \Re\left\{\alpha_{\mathrm{p}}\right\} \nabla I(\mathbf{r}) + \frac{n_{\mathrm{m}}}{c} \sigma_{\mathrm{ext}} I(\mathbf{r}) \hat{\mathbf{k}} \tag{24}$$

where $I(\mathbf{r}) = \frac{1}{2}n_{\rm m}c|\mathbf{E}(\mathbf{r})|^2$ is the intensity of the electric field, $\hat{\mathbf{k}}$ is the unit vector defining propagation direction, and we have not considered spin-dependent terms [15–17] that are generally negligible in optical trapping with Gaussian beams.

The first term in Eq. (24) represents the gradient force and is responsible for particle confinement in optical tweezers. Arising from the potential energy of a dipole immersed in the electric field, it is conservative. Particles with a positive $\Re\{\alpha_{\rm p}\}$ will be attracted toward the high intensity region of the optical field. Conversely, when the real part of the polarizability is negative the particles are repelled by the high intensity region. The second term in Eq. (24) is the scattering force. It is responsible for the radiation pressure and is non-conservative. Furthermore, it is directed along the propagation direction of the laser beam [5].

In our experiments we have an incident paraxial laser beam with power P_i, and a typical Gaussian intensity profile, which propagates along the z axis:

$$I(r,z) = I_0\left[\frac{w_0}{w(z)}\right]^2 \exp\left[\frac{-2r^2}{w^2(z)}\right]. \tag{25}$$

Thus, we can define the trap stiffnesses, κ_r, related to the gradient force in the transverse (radial) plane, and κ_z, along the direction of propagation [18]:

$$\kappa_r = 2\frac{\Re\{\alpha_{\rm p}\}}{cn_{\rm m}}\frac{I_0}{w_0^2}, \qquad \kappa_z = \frac{\Re\{\alpha_{\rm p}\}}{cn_{\rm m}}\frac{I_0}{z_0^2}. \tag{26}$$

where $I_0 = 2P_i(\pi w_0^2)^{-1}$ is the maximum intensity at the focus of the beam, w_0 is the Gaussian beam waist and $z_0 = n_{\rm m}\pi w_0^2\lambda_0^{-1}$ is the beam Rayleigh range [7]. This beam parameters can be estimated by considering a diffraction limited focus, so that the paraxial Gaussian beam waist w_0 is provided by the Abbe criterion [19], $w_0 = 0.5\lambda_0/\mathrm{NA}$. For small particles the size scaling of the trap stiffnesses increases with a cubic power law, $\kappa \propto R^3$, in agreement with the volumetric scaling of the particle polarizability in the dipole approximation. The comparison between values calculated in dipole approximation and by T-matrix methods is very good up to $R \approx 200$ nm [9].

Thus, the power extinct by a small particle in the intracavity trap can be estimated as $P_{\rm ext} \approx I(r,z)\sigma_{\rm ext,d}$, that for a non-absorbing particle reduces to $P_{\rm ext} \approx I(r,z)\sigma_{\rm scat,d}$. Hence we can estimate the particle's optical loss, $l_{\rm scat} \approx I(r,z)\sigma_{\rm scat,d}/P_i$, that regulates the operation of the intracavity trap. First, we consider the optical loss in terms of particle's

displacement that in the dipole approximation has a simple analytical expression:

$$l_{\text{scat}}(r,z) = \frac{2\sigma_{\text{scat,d}}}{\pi w_0^2}\left[\frac{w_0}{w(z)}\right]^2 \exp\left[\frac{-2r^2}{w^2(z)}\right]. \tag{27}$$

The optical loss has a profile that follows the Gaussian intensity of the laser beam and has a maximum at the beam focus, when the particle's scattering is maximum.

Thus, we can consider the size scaling behaviour of the optical loss when the particle is trapped in proximity of the focal spot, i.e., for maximum scattering:

$$l_{\text{scat}} = \frac{2\sigma_{\text{scat,d}}}{\pi w_0^2} \approx \frac{8\sigma_{\text{scat,d}}\text{NA}^2}{\pi\lambda_0^2}. \tag{28}$$

Note how this size scaling is valid only for particles smaller than the laser wavelength, λ_0, and it is regulated by the ration between the scattering cross section with respect to the wavelength squared. Hence, the smaller the particle the less effective the intracavity trapping. For a dielectric non-absorbing particle the size scaling of the optical losses is rapidly decreasing as the scattering cross section, *i.e.*, $l_{\text{scat}} \sim R^6$. Instead, for metal absorbing particles, e.g., gold nanoparticles, the size scaling of the optical losses is linked to the extinction cross section (that includes absorption) and hence follows a volumetric decrease. However, in this latter case, increased absorption can have a detrimental effect on the stability of the trap resulting in increased radiation pressure and heating effects.

These expressions have been used to obtain the plots shown in Supplementary Figure 6 where we show the optical loss, $l_{\text{scat}}(r,z)$, in the intracavity trap for our experimental parameters as a function of particle's displacement (Supplementary Figure 6(a)), and the size scaling dependence of the optical loss at the focus as a function of particle's radius for a polystyrene spherical particle in water (Supplementary Figure 6(b)). Despite the dipole approximation can be safely applied only for a particle's radius below 200 nm, we can observe how large particles are needed to increase the scattering in the trap and increase the cavity optical losses to efficiently operate the intracavity feedback trapping. This clearly shows that, for our experimental parameters, intracavity trapping is efficient at the microscale while reduces to a standard single-beam optical trapping at the nanoscale. However, the intracavity optical trapping approach can in principle be scaled down to particles significantly smaller than the wavelength by changing the experimental parameters. In particular, as shown in Eq. (28), increasing the numerical aperture would allow to increase the losses due to the particle and therefore make the intracavity optical trapping more efficient at the nanoscale.

SUPPLEMENTARY NOTE 6

Temporal dynamics of fiber laser, intracavity trapping, and Brownian fluctuations

Different temporal dynamics can be identified in the intracavity trapping scheme. These dynamics correspond to three temporal scales: fast laser dynamics (ns), intracavity optical trapping dynamics (ms), Brownian dynamics at equilibrium (s). As we discussed before in supplementary Note 1 and showed in Supplementary Figures 2 and 3, the Brownian dynamics of the trapped particle in the intracavity optical tweezers is of several seconds, related to the low intensity regime of the laser operation at equilibrium. Instead, the time scale of nonlinear intracavity trapping can be measured by tracking the position of a particle entering the trap region. This is shown in Supplementary Figure 7 where we show the trapping dynamics of a 4.9-µm-diameter polystyrene particle (Supplementary Figure 7(a)) and the corresponding power drop (Supplementary Figure 7(b)). The particle falls to the trap and power drops in 100 ms. Indeed, the nonlinear feedback that regulates the trapping dynamics occurring in this temporal regime is two orders of magnitude faster than the typical Brownian dynamics measured by autocorrelation functions or PSD. Finally, the dynamics of the laser is much faster than the trapping dynamics. The response time of the laser feedback is approximated by the round trip time divided by the losses. With a fair approximation, the response time of the laser to any dynamical change can be described as the photon lifetime in the cavity:

$$\tau = \frac{t_{\text{round trip}}}{(1 - l_{\text{total}})}, \tag{29}$$

where $t_{\text{round trip}} = nL/c$ is the time taken for a photon to make one round-trip in a cavity with an optical length nL, with n and L refractive index of the propagation medium and cavity length, and l_{total} represents the total optical loss of the cavity in a round-trip. For our experimental system we have that the total fiber lengths is 3.5 m, its refractive index is 1.5, the free space length is 50 cm, hence the round trip time is about 19 ns. The calculated optical loss of the cavity including all the different parts, the Fresnel reflection losses of the components, and collimator-collimator coupling loss, is about 50%. Therefore, the calculated photon life time and hence the response time of the cavity is about 38 ns. This nanosecond time scale of the laser dynamics compared to millisecond time scale of the trapping ensures an efficient feedback control of the trapping dynamics. To measure the response of the laser cavity to a dynamical loss, we replace the trapping optics by a Fiber-

coupled acousto-optic modulators (AOM) (Gooch and Housego, 200 MHz bandwidth). The transmission of the AOM and then the optical loss of the cavity is varied by an arbitrary function generator (AFG) (Tektronix-3242, 240 MHz bandwidth). The optical signal from the 1% port of the 99:1 coupler is detected with an InGaAs photodiode (EOT-3010, 1.5 GHz bandwidth) connected to an oscilloscope (LeCroy-62Xs, 600 MHz bandwidth). To apply optical loss, the constant DC voltage applied to the AOM by the AFG drops to zero in less than 10 ns. Supplementary Figure 7(c) shows the response of the laser cavity. The $10-90\%$ response time is about 20 ns.

SUPPLEMENTARY REFERENCES

[1] Woerdemann, M., Berghoff, K., and Denz, C. Dynamic multiple-beam counter-propagating optical traps using optical phase-conjugation. *Opt. Express* **18**, 22348–22357 (2010).

[2] Pitzek, M., Steiger, R., Thalhammer, G., Bernet, S., and Ritsch-Marte, M. Optical mirror trap with a large field of view. *Opt. Express* **17**, 19414–19423 (2009).

[3] Juan, M. L., Gordon, R., Pang, Y., Eftekhari, F., and Quidant, R. Self-induced back-action optical trapping of dielectric nanoparticles. *Nat. Phys.* **5**, 915–919 (2009).

[4] Descharmes, N., Dharanipathy, U. P., Diao, Z., Tonin, M., and Houdré, R. Observation of backaction and self-induced trapping in a planar hollow photonic crystal cavity. *Phys. Rev. Lett.* **110**, 123601—1–4 (2013).

[5] Ashkin, A. Acceleration and trapping of particles by radiation pressure. *Phys. Rev. Lett.* **24**, 156–159 (1970).

[6] Donato, M. G., Brzobohatỳ, O., Simpson, S. H., Irrera, A., Leonardi, A. A., Lo Faro, M. J., Svak, V., Maragò, O. M., and Zemánek, P. Optical trapping, optical binding, and rotational dynamics of silicon nanowires in counter-propagating beams. *Nano. Lett.* **19**, 342–352 (2019).

[7] Jones, P. H., Maragò, O. M., and Volpe, G. *Optical Tweezers: Principles and Applications.* Cambridge University Press, (2015).

[8] Pesce, G., Volpe, G., Maragò, . M., Jones, P. H., Gigan, S., Sasso, A., and Volpe, G. Step-by-step guide to the realization of advanced optical tweezers. *J. Opt. Soc. Am. B* **32**, B84–B98 (2015).

[9] Polimeno, P., Magazzù, A., Iatì, M. A., Patti, F., Saija, R., Esposti Boschi, C. D., Donato, M. G., Gucciardi, P. G., Jones, P. H., Volpe, G., and Maragó, O. M. Optical tweezers and their applications. *J. Quant. Spectrosc. Radiat. Transfer* **218**, 131–150 (2018).

[10] Nieminen, T. A., Stilgoe, A. B., Heckenberg, N. R., and Rubinsztein-Dunlop, H. Approximate and exact modeling of optical trapping. In *Optical Trapping and Optical Micromanipulation VII*, volume 7762, 77622V—1–8. International Society for Optics and Photonics, (2010).

[11] Gordon, J. P. Radiation forces and momenta in dielectric media. *Phys. Rev. A.* **8**, 14–21 (1973).

[12] Chaumet, P. and Nieto-Vesperinas, M. Time-averaged total force on a dipolar sphere in an electromagnetic field. *Opt. Lett* **25**, 1065–1067 (2000).

[13] Arias-González, J. R. and Nieto-Vesperinas, M. Optical forces on small particles: attractive and repulsive nature and plasmon-resonance conditions. *J. Opt. Soc. Am. A* **20**, 1201–1209 (2003).

[14] Draine, B. T. and Goodman, J. Beyond clausius-mossotti-wave propagation on a polarizable point lattice and the discrete dipole approximation. *Astrophys. J.* **405**, 685–697 (1993).

[15] Gao, D., Ding, W., Nieto-Vesperinas, M., Ding, X., Rahman, M., Zhang, T., Lim, C., and Qiu, C. W. Optical manipulation from the microscale to the nanoscale: fundamentals, advances and prospects. *Light Sci. Appl.* **6**, e17039—1–15 (2017).

[16] Albaladejo, S., Marqués, M. I., Laroche, M., and Sáenz, J. J. Scattering forces from the curl of the spin angular momentum of a light field. *Phys. Rev. Lett.* **102**, 113602—1–4 (2009).

[17] Marqués, M. I. and Sáenz, J. J. Reply to comment on scattering forces from the curl of the spin angular momentum of a light field. *Phys. Rev. Lett.* **111**, 059302 (2013).

[18] Zemánek, P., Jonáš, A., Jákl, P., Ježek, J., Šerỳ, M., and Liška, M. Theoretical comparison of optical traps created by standing wave and single beam. *Opt. Commun.* **220**, 401–412 (2003).

[19] Born, M. and Wolf, E. *Principles of optics: Electromagnetic theory of propagation, interference and diffraction of light.* Cambridge University Press, (1999).
